# Second brazilian consensus on the treatment of advanced prostate cancer: a SBOC-SBU-SBRT panel review

**DOI:** 10.1590/S1677-5538.IBJU.2018.0798

**Published:** 2019-07-27

**Authors:** Andre Deeke Sasse, Rodolfo Borges dos Reis, Lucas Mendes Nogueira, Fernando Cotait Maluf, Daniel Herchenhorn, Oren Smaletz, Volney Soares Lima, Fábio Schutz, Diogo Bastos, Evanius Garcia Wiermann, Igor Alexandre Protzner Morbeck, Leonardo Fontes Jardim, Vinicius Carrera Souza, Icaro Thiago Carvalho, Elton Trigo Teixeira Leite, Archimedes Nardozza, Antonio Carlos Lima Pompeo, Francisco Bretas, Marcos Lima de Oliveira Leal, Marcus Vinicius Sadi, José Ricardo Tuma da Ponte, Gustavo F. Carvalhal

**Affiliations:** 1 Grupo SONHE, Oncologia, Campinas, SP, Brasil; 2 Departamento de Urologia, Universidade de São Paulo- USP, Campus de Ribeirão Preto, Ribeirão Preto, SP, Brasil; 3 Divisão de Urologia e Departamento de Cirurgia Hospital das Clínicas, Universidade Federal de MG - UFMG, Belo Horizonte, MG, Brasil; 4 Hospital Beneficência Portuguesa de São Paulo, SP, Brasil; 5 Rede D'Or São Luiz, Rio de Janeiro, Brasil; 6 Departamento de Oncologia, Hospital Israelita Albert Einstein, São Paulo, Brasil; 7 Oncocentro, Belo Horizonte, MG, Brasil; 8 Departamento de Oncologia, Instituto do Câncer do Estado de São Paulo - ICESP, SP, Brasil; 9 Sociedade Brasileira de Oncologia Clinica - SBOC, Belo Horizonte, MG, Brasil; 10 Clinica AMO, Salvador, Bahia, Brasil;; 11 Hospital Sírio-Libanês, São Paulo, SP, Brasil; 12 Departamento de Urologia e Cirurgia, Universidade Federal de São Paulo - UNIFESP, São Paulo, SP, Brasil; 13 Faculdade de Medicina do ABC, Urologia, Santo André, SP, Brasil; 14 Hospital Mater Dei, Belo Horizonte, MG, Brasil; 15 Departamento de Urologia, Universidade Federal da Bahia - UFBA, Salvador, Bahia, Brasil; 16 Departamento de Urologia, Universidade do Estado do Pará - UEPA, Belém, PA, Brasil; 17 Divisão de Urologia e Departamento de Cirurgia, Pontificia Universidade Católica do Rio Grande do Sul – PUC RS, Porto Alegre, RS, Brasil

**Keywords:** Prostatic Neoplasms, Therapeutics, Consensus

## Abstract

Prostate cancer is the second most common cancer and the fifth leading cause of cancer deaths. In Brazil, it is likewise the second most common cancer among men, second only to non-melanoma skin cancers.

The aim of this consensus is to align different opinions and interpretations of the medical literature in a practical and patient-oriented approach. The first Brazilian Consensus on the Treatment of Advanced Prostate Cancer was published in 2017, with the goal of reducing the heterogeneity of therapeutic conduct in Brazilian patients with metastatic prostate cancer. We acknowledge that in Brazil the incorporation of different technologies is a big challenge, especially in the Sistema Único de Saúde (SUS), which allows for the disparity in the options available to patients treated in different institutions. In order to update the recommendations and to make them objective and easily accessible, once more a panel of specialists was formed in order to discuss and elaborate a new Brazilian Consensus on Advanced Prostate Cancer. This Consensus was written through a joint initiative of the Brazilian Society of Clinical Oncology (SBOC) and the Brazilian Society of Urology (SBU) to support the clinical decisions of physicians and other health professionals involved in the care of patients with prostate cancer.

## INTRODUCTION

The first Brazilian Consensus on the Treatment of Advanced Prostate Cancer was published in 2017, (1) with the goal of reducing the heterogeneity of therapeutic conduct in Brazilian patients with metastatic prostate cancer. The incorporation of different technologies is a challenge in Brazil, especially in the Sistema Único de Saúde (SUS), which allows for the disparity in the options available to patients treated in different institutions. To update and provide objective, easily accessible recommendations, a panel of specialists was formed once more to discuss and elaborate a new Brazilian Consensus on Advanced Prostate Cancer.

Prostate cancer is the second most common cancer among men and the fifth leading cause of cancer deaths in the World. In Brazil, it is likewise the second most common cancer among men, second only to non-melanoma skin cancers (2), with an incidence of 68.220 new cases per year, and an estimated rate of 13.772 annual deaths (3).

A variety of new systemic treatments were instituted for the treatment of both hormone-sensitive and castrate-resistant metastatic prostate cancer (mCRPC) since the approval of docetaxel as a first line therapy for mCRPC in 2004. Since the pivotal studies that led to the approval of the new therapies are for the most part contemporary, direct comparisons between treatments are lacking. Additionally, studies about the different sequencing of treatments are rare. Therefore, decisions regarding the best treatment options basically rely on access to treatments, critical appraisal of the literature, and physician's experience. The aim of this Consensus is to align different opinions and interpretations of the medical literature in a practical and patient-oriented approach.

## OBJECTIVE

This Consensus was written through a joint initiative of the Brazilian Society of Clinical Oncology (SBOC) and the Brazilian Society of Urology (SBU) to support the clinical decisions of physicians and other health professionals involved in the care of patients with prostate cancer.

The manuscript is targeted mainly to clinical oncologists, urologists and radiotherapists.

## MATERIALS AND METHODS

The SBOC and the SBU formed a panel of 23 specialists from different regions of Brazil, based on their scientific prominence and clinical experience in the care of patients with prostate cancer. A moderator (clinical oncologist), 10 other clinical oncologists, 10 urologists, and two radiation oncologists participated of the present consensus. Forty clinically relevant questions concerning the care of men with advanced prostate cancer were previously selected by a subgroup of the participants for a panel discussion. The questions were created aiming at the treatment and follow-up of patients with recurrent or metastatic prostate cancer. Aspects related to epidemiology, screening, diagnosis, prognosis, and treatment of localized disease were not discussed in this consensus.

Following elaboration of the questions, they were distributed and forwarded to the members of the panel to produce answers based on the critical analysis and systematic review of the published literature. Participants were encouraged to only refer to studies with a solid methodology and with predefined levels of scientific evidence. The initial answers were forwarded to the moderator of the consensus to be written in a uniform style, and were subsequently forwarded to all of the participants for a preliminary evaluation.

The panel of the Second Brazilian Consensus on the Treatment of Advanced Prostate Cancer was held on with all participants March 22, 2017, in São Paulo, Brazil, during the VIII International Meeting of Urologic Oncology. A modified Delphi methodology was employed to obtain consensus (4). Each answer was discussed and voted by all Consensus participants in loco. When a participant was not experienced enough to vote for an answer or to choose a valid option, or even when he/she had conflicts of interest, the option “It does not concern to my practice/I'd rather not vote” was selected. In these cases, these votes did not count as valid for the consensus on that answer. A consensus on a specific answer was reached whenever 2/3 of the participants agreed upon it. In cases in which the consensus was not achieved on a first voting session, the panel discussed the conflicting points under the guidance of the moderator. Subsequently, a second vote was held. If a consensus was not reached again, it was stated in the manuscript that there was not a consensual agreement on a specific answer. The final manuscript was written based on recording of the meeting, and subsequently approved by all of the participants of the panel. Our sponsor provided unconditional financial and educational support through SBOC for the preparation and conduct of the meeting, without any influence in the votes and their content. No participant received any fee or financial incentive to participate in the voting process.

## RESULTS

### Consensus Development and Discussions

**Figure 1:**
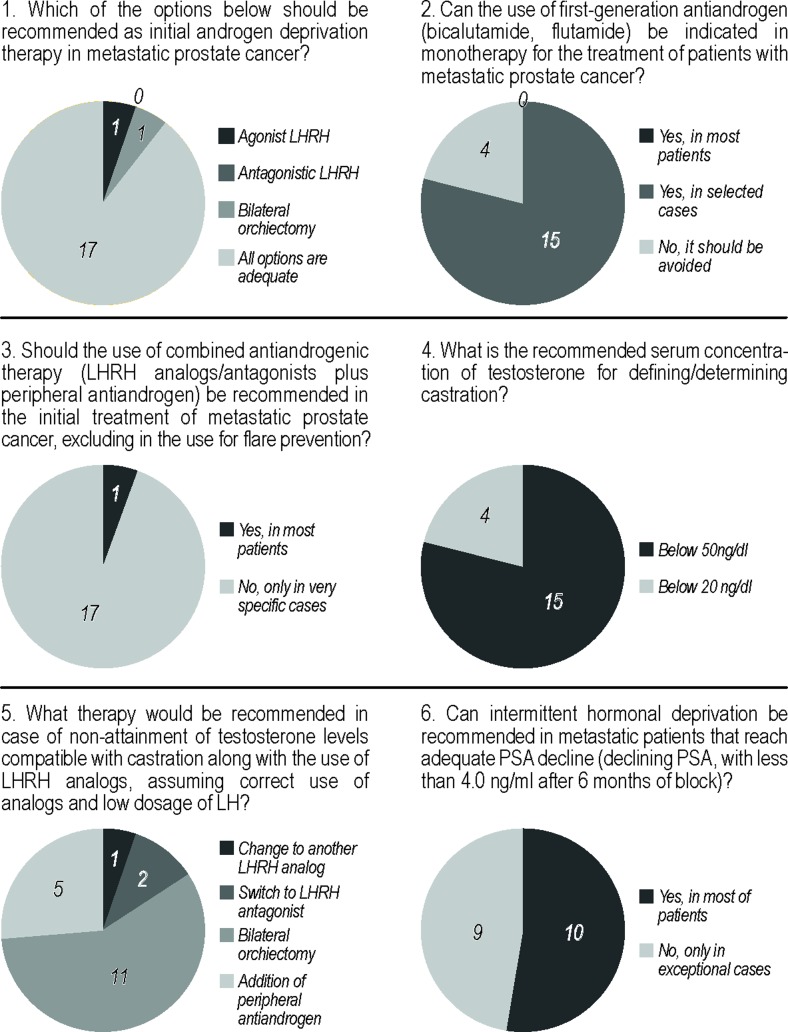

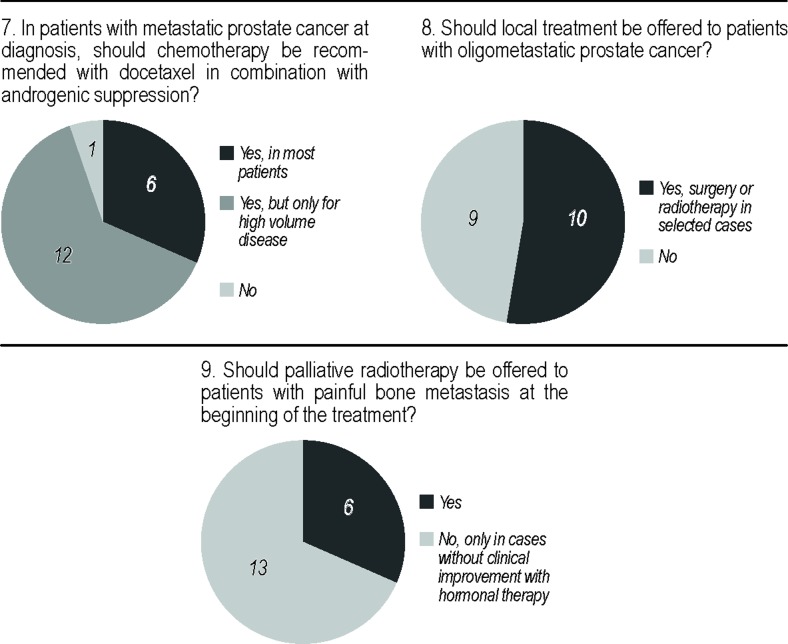
Summary of all results of the voting process, with or without consensus.

The final results of the voting process, with or without consensus, are summarized in Figure-1. The main resolutions and recommendations of the Second Brazilian Consensus on the Treatment of Advanced Prostate Cancer are discussed below.

### Hormone-sensitive Metastatic Prostate Cancer

Prostate cancer is associated with an elevated disease-specific mortality when metastatic in spite of being stereotyped as an indolent disease. As cellular proliferation in this disease state is highly dependent on the androgen pathway, the main goal of initial hormonal therapy is to block androgen activity, avoiding the signalization to cell proliferation in the hormone sensitive cells (5). Androgen deprivation therapy (ADT) is the initial treatment strategy, and the panel recommended with 89% of votes that either 1) agonists or 2) antagonists of luteinizing hormone-releasing hormone (LHRH) or 3) bilateral orchiectomy are adequate options to initial systemic therapy (question 1). These three options are equally effective as androgen deprivation therapies in metastatic prostate cancer, and are more effective than monotherapy with antiandrogens (6). When LHRH agonists are preferred, one should use an antiandrogen before or concomitant with an LHRH agonist for at least 7 days in patients at risk of complications due to the transient rise in testosterone levels in these situations (testosterone flare) (6). Recent studies have suggested that orchiectomy may lead to less osteoporotic, cardiac, and vascular complications than LHRH agonists (7).

The panel also considered that monotherapy with first generation antiandrogens (e.g., bicalutamide) may be indicated in select patients. It is not the gold standard, but should not be contraindicated (question 2). The use of high doses of bicalutamide (150mg q.d.) in this setting was compared with orchiectomy in a randomized study, with similar clinical outcomes (8).

The use of combined androgen blockade (LHRH agonist/antagonist plus peripheral antiandrogens) in the initial treatment of metastatic prostate cancer was not recommended by the panel as a routine, but was recommended in select cases by 94% (question 3). A systematic review with meta-analysis of 20 studies and 6871 patients has shown that the net benefit of a “complete blockade” is clinically irrelevant (9).

As recommended in the First Brazilian Consensus (1), there was consensus that testosterone levels below 50ng/dl should be used as the definition of effective castration, despite the existing literature suggesting a potential clinical benefit of lower levels or testosterone (below 20ng/dl) (10) (question 4). For patients initially treated with LHRH agonists who in spite of low levels of LH remain with testosterone levels above castration levels, 58% of the panel recommended surgical castration; however, there was no consensus, since other options could be viable (e.g., change of LHRH agonist, LHRH antagonist, addition of an antiandrogen) (question 5).

The use of intermittent hormone blockade was studied in various randomized trials as a strategy to allow for intervals of normal testosterone levels with benefits in quality of life, less cardiovascular risk, and less lean mass deterioration,. None has shown clinically significant benefits in quality of life, and intermittent hormone blockade was not shown to be non-inferior to continuous blockade regarding overall survival (11, 12). Thus, the panel did not reach onsensus on the recommendation of intermittent hormonal blockade in metastatic patients in men who achieve adequate declines in PSA levels (declining PSA, < 4ng/ ml after 6 months of blockade). With 53% of the votes, intermittent hormonal blockade was recommended for most patients; however, 47% of the panel voted that it could be recommended only in exceptional cases (question 6).

Regarding the use of chemotherapy for patients with metastatic prostate cancer at the diagnosis (de novo), the panel reached consensus in recommending the use of docetaxel in combination to ADT, but only in patients with high volume disease (question 7). Randomized studies have shown a clinically important and statistically significant benefit in using docetaxel in conjunction with ADT compared with ADT alone (13). However, recent data from the study CHAARTED suggest that these benefits are restricted to patients with high volume of disease at diagnosis (14).

Subsequent to the Consensus meeting and votes, the LATITUDE and STAMPEDE studies were published, in which similar benefits were shown regarding response rates, progression-free survival and overall survival with ADT plus abiraterone vs. ADT alone (15, 16). Therefore, abiraterone is also an option in this setting.

Due to the elevated risk of symptoms related to disease progression in the pelvis, there are ongoing trials studying local therapies such as radiotherapy or surgery for men with low volume metastatic disease and a long life expectancy (17). However, there was no consensus about offering local treatments to patients with oligometastatic disease at diagnosis, with 53% of the panelists recommending local treatment, and 47% of panelists contraindicating (question 8).

Furthermore, the panel suggested with 68% of the votes that radiotherapy for pain control should not be offered in the beginning of treatment for the patient with metastases, reserving it for those cases that do not achieve pain control with hormonal treatment (question 9).

### Castrate-resistant Prostate Cancer (CRPC)

The patient with castrate-resistant prostate cancer (CRPC) generally carries a worse prognosis, with a considerable risk of deterioration of his quality of life and an elevated mortality. However, in this stage, the disease is also highly heterogeneous, with various phenotypes, biologic characteristics and clinical outcomes (18). It is defined as a prostate cancer that progresses biologically, clinically or on imaging studies in the face of castrate testosterone levels (< 50ng/ml). Many proposed mechanisms explain the development of androgen resistance, and strategies are being developed to overcome this problem (19). There was a consensus among panelists (79%) that there is no need to add an antiandrogen to the prevailing androgen deprivation therapy to define hormone resistance (question 10).

In patients with biochemical progression but a negative bone scan and an abdominal/pelvic computerized tomography (CT)/magnetic resonance imaging (MRI) and without clinical evidence of disease, it was recommended by 74% of the panel that no other diagnostic method is required (question 11). Also, it was defined that for M0 CRPC patients in biochemical recurrence, no additional therapy is required (question 12). In spite of this recommendation, after the Consensus meeting, the PROSPER and SPARTAN studies were published, which showed a clear benefit of the early use of enzalutamide and apalutamide in this clinical setting, eventually changing the opinion of the panel. The panel also recommended the screening for metastases in asymptomatic patients with regular imaging studies, depending on the PSA doubling time (PSADT) (question 13).

No significant benefit was ever demonstrated with vintage endocrine manipulations (e.g., bicalutamide, flutamide, DES, estramustine, ketoconazole). Case series have only shown isolated responses with a drop in serum levels of PSA, however with no gain in overall survival or in long-term disease control (20). The panel was of the consensus that for men with asymptomatic or oligosymptomatic metastatic CRPC, it is inadequate to opt for vintage endocrine therapies if abiraterone or enzalutamide are available (question 14). Nonetheless, as access to these drugs is restricted, especially in the public health system, these vintage manipulations were considered appropriate if neither abiraterone nor enzalutamide are available (question 15). Abiraterone and enzalutamide were recommended as first-line treatments in addition to conventional ADT for metastatic CRPC in asymptomatic or mildly symptomatic men by 90% of the participants (question 16). There was no consensus regarding the indication of docetaxel in this situation (question 17).

In symptomatic, metastatic CRPC, 75% of the specialists recommended abiraterone or enzalutamide as a first line of treatment (pre-chemotherapy) in addition to ADT (question 18), a situation in which docetaxel can also be considered a standard. Although the studies that led to the approval of abiraterone (21) and enzalutamide (22) in metastatic CRPC pre-docetaxel included mostly asymptomatic or mildly symptomatic men, the extrapolation of the benefits to patients with more advanced disease was considered adequate. Finally, 95% of the panelists voted that abiraterone or enzalutamide should be the preferred recommendation for metastatic CRPC in asymptomatic patients, if all therapeutic options were available (question 19).

There was much debate about clinical and laboratorial criteria to help select the first line and the best sequence of treatments. The Gleason score was not recommended as a valid tool to decide between chemotherapy, abiraterone or enzalutamide (question 20), in spite of the fact that a higher Gleason score is usually associated with a poorer prognosis. There was also no consensus regarding the importance of the time of response to conventional ADT in choosing between abiraterone/enzalutamide or chemotherapy as a first line therapy for metastatic CRPC (question 21). The presence of visceral metastases was considered an important factor in a choice of chemotherapy over abiraterone/enzalutamide (question 22).

The panel voted with 70% consensus that one should not offer retreatment with docetaxel in men who already received docetaxel, except in select few cases or in the case of lack of access to the other therapies (question 23).

Primary resistance to abiraterone or enzalutamide was defined as clinical or radiographic progression within 3 months of the beginning of therapy (question 24). In such a case, early discontinuation of treatment would be justified. One has to consider, however, the possibility of flare and of PSA changes that do not necessarily reflect disease progression.

There was no consensus regarding the maximal duration of treatment with docetaxel in patients with metastatic CRPC. Although the most important studies suggest 10 cycles of chemotherapy (23), some specialists suggest that one should observe limiting toxicities and the impact on the patient's quality of life to define when to interrupt/stop therapy (question 25).

### Staging and Treatment Monitoring

The panel recommended the performance of new staging exams whenever a new line of treatment is introduced in men with metastatic CRPC (question 26). On the other hand, the performance of routine imaging studies to evaluate the risk of fractures was not recommended to all men with bone metastases detected in bone scans (question 27). In these situations, clinical evaluation on the risk of fractures would be important, especially in situation of disease progression.

Isolated PSA progression, without clinical or radiographic progression, should not be considered a strong enough reason to modify treatment in men with CRPC, with 95% agreement (question 28). Unanimously, the panel recommended that treatment monitoring should be performed periodically with PSA and imaging studies (question 29).

### Bone Therapies

It is important to identify men with an elevated risk of symptomatic events related to bone, such as pathological fractures, hypercalcemia, and opioid analgesia for pain in CRPC, since these events are associated with increased mortality and decreased quality of life. There was consensus in that inhibitors of osteolysis (e.g., zoledronic acid, denosumab) should be indicated to men with metastatic CRPC and bone disease. However, there is no evidence of benefit of such therapies in hormone-sensitive disease, even in the setting of a high volume of metastases (question 30) (24). The panel also agreed that there is no indication for the use of pamidronate or clodronate as bone therapy for metastatic CRPC (question 31). It is important to avoid monthly administration of such drugs for more than two to three years, due to the increased risk of osteonecrosis of the jaw (25).

The panel was unanimous in contraindicating the use of radium-223 in asymptomatic metastatic CRPC (question 32). However, 85% of the specialists recommended that radium-223 should be reserved for men with symptomatic, non-visceral bone metastases in CRPC (question 34). It is indicated either before or after docetaxel (question 33) and even with abiraterone or enzalutamide (question 35). After the Consensus meeting, the publication of results from the ERA-223 study analysing the combination of radium-223 and abiraterone revealed that there was an increased rate of fractures and deaths with the combined treatment, and many regulatory agencies contraindicated this approach. Therefore, the panel recommends extreme caution when considering the combination of radium-223 and the new hormonal treatments.

### Sequencing of Available Treatments

As the ideal sequence of therapies is still unknown, strategies were discussed. The panel recommended that in the case of disease progression during treatment with abiraterone/enzalutamide the sequential use of one of these agents (abiraterone/enzalutamide) is not adequate due to the elevated risk of cross-resistance (question 36).

At the moment, 95% of the specialists agreed that the use of biomarkers such as AR-V7 to select between abiraterone/enzalutamide or chemotherapy is still not ready for clinical use (question 37).

The use of platin analogues along with docetaxel was not recommended (question 38).

Finally, the specialists evaluated the treatment recommendation for men with an initial response to docetaxel, but with subsequent disease progression. There was no consensus if progression occurred in less than three months after the discontinuation of docetaxel, however in the case of disease progression after three months, the initiation of abiraterone or enzalutamide was recommended (questions 39 and 40). This is supported by the randomized studies COU-AA-301 (26) and AFFIRM (22). The net benefit found in these studies reaffirmed the importance of the androgen receptor in CRPC. Therefore, if the patient has not been exposed to abiraterone or enzalutamide in previous treatments, the preference should be one of these agents. Alternatively, there are randomized studies supporting the use of cabazitaxel (TROPIC) (27), radium-223 (ALSYMPCA) (28) and retreatment with docetaxel (PRINCE). Other factors such as availability, access, toxicity profile, and clinical and pathological characteristics should be considered in the definition of the sequential line of treatment after docetaxel failure.

## DISCUSSION

The main goal of the treatment of patients with advanced prostate cancer should be to increase survival while maintaining quality of life. Since metastatic prostate cancer is an incurable disease, therapeutic strategies should prioritize the maximum time of cancer control with the minimum associated toxicity. The treatment of metastatic prostate cancer is complex due to the multitude of available therapies, all with different response and toxicity profiles. Additionally, it is a disease that typically affects older men, and the existence of comorbidities increases the challenge in the choice of therapy. It is also a heterogeneous disease with a lack of direct comparisons between many available treatments.

Many times, we chose to refer to Abiraterone and Enzalutamide as a single option, perhaps giving the impression they are very similar in properties and mode of action. In fact, even considering the differences between Enzalutamide and Abiraterone, the evidence supporting the indication of the two options was considered similar. More important, existing evidence is unable to answer a very important sequencing question, of whether enzalutamide followed by abiraterone is better than abiraterone followed by enzalutamide.

Acknowledging how rapidly new evidence is added to the literature and knowing that the applicability of the newer information is always a great challenge, this Consensus aims to provide valuable information on treatments to help guide and adapt the new information to our reality in Brazil.

The choice of therapies must be individualized depending on patient characteristics, with some options preferred in many clinical settings.

The specialist's panel has worked on recommendations for the preferred therapies, on the indications that justify the change of therapies, and on some of the available strategies to better select the sequencing of treatments to maximize disease control with the available therapeutic arsenal.

The lack of consensus on some topics indicates a lack of strong evidence supporting some clinical decisions. In creating the recommendations, we considered the potential benefits, the availability, the costs, as well as adverse events and the risks involved.

The recommendations herein contained should be seen as a guide to clinical conduct. It is important to stress that the adherence to the recommendations does not guarantee a satisfactory clinical outcome. The physician should make the final decision about the most adequate treatment strategy after discussing the available treatment options with the patient. However, it is recommended that significant deviations from the suggested practical conducts be justified, with well-documented reasons.
